# St. George's Hospital

**Published:** 1830-04-01

**Authors:** 


### CLINICAL REVIEW.

### XII.

### ST. GEORGE'S HOSPITAL.

UMBILICAL HERNIA—"ASTHMA"—DROPSY—OPERATION—DEATH—ENORMOUS HYPERTROPHY OF THE HEART.

The following case which occurred very lately at this hospital is interesting alike to the surgeon and the physician, and very important in a practical point of view.

*Case.* Thomas Clark, æt. 60, a publican residing at Knightsbridge, admitted Jan. 4, 1830, under the care of Mr. Keate.

Present symptoms are ;—dyspnœa—orthopnœa — cough in "asthmatic" paroxysms—no pain in chest—no palpitations of the heart—occasional flatulence—œdema of lower extremities — body very fat — face bloated, and somewhat anxious—mind rather confused. Pulse regular, slightly jarring ; skin warm ; tongue moist, whitish ; bowels not open this morning ; urine very scanty.

Belly of enormous size, chiefly from fat, with an indistinct sense of fluctuation. At the umbilicus is a protrusion nearly six inches in diameter every way, most prominent at the upper part and right side, flattened in the centre in the situation of the navel and for a circular space about two inches in diameter. It is generally soft, with harder portions, and has all the characters of an umbilical hernia, partly intestinal and partly omental. Integuments not discoloured, except in the circular flattened space alluded to, where they are brown from the long continued pressure of a truss. Tumour slightly painful on pressure, which occasions a sort of guggling within it, but does not in any degree reduce its bulk. Some little tenderness on pressure of the abdomen in the neighbourhood.

In the right groin a large scrotal hernia, chiefly, if not entirely, intestinal, and easily reduced ; besides this, a slight hydrocele of the tunica vaginalis on this side.

On examining chest with stethoscope the lower part of either lung presents a very imperfect respiratory sound, with a small sibilant *râle ;* respiration in upper parts of chest attended with louder and more mucous sound than natural. Heart's action attended with more impulsion than there should be, but the excessive quantity of fat and the noisy respiration obscure the cardiac indications. No bruit above right clavicle, or evidence of dilatation of the aorta.

Patient says he has laboured under the "asthma" since August last, but the friends affirm that he has suffered from it in a mild degree for eighteen months. Has had the orthopnœa, more or less, since September, but only to a distressing degree for a week or two. Scrotal hernia has existed as long as he can recollect—the umbilical about 16 years. Has worn a truss for the latter, but been unable to reduce it entirely for some time. A week ago it was the size of an orange, and he procured a fresh truss from Salmon and Ody. Has passed his motions up to within the last 36 hours, but they seem to have been scanty. For the period mentioned he has had no stool, and has been treated for strangulated hernia by bleeding ; castor oil, which he vomited ; and an enema exhibited at 3 o'clock this morning, which has not yet returned. Previous to these hernial symptoms he has enjoyed pretty good health, with the exception of occasional attacks of gout and his asthmatic complaint. He has eat and drank immensely.

*V. S. ad syncopen (in upright posture.)*

After the bleeding, which the patient bore well, the taxis was carefully employed by the house-surgeon, Mr. Smith, but without making the least impression on the tumour. At 5 p. m. the surgeons again met in consultation. The tumour was now larger and somewhat more painful ; the patient also complained of a little more pain in the abdomen, and had had no stool. It was determined to administer the tobacco glyster, which was done under the direction of Mr. Smith, with the effect of producing considerable but not alarming faintness, and violent vomiting of dark brown matters, apparently stercoraceous. The taxis was again employed with the same ill success as before.

At 9, p. m. the surgeons met again. The tumour was now extremely tense and tender upon pressure, but the integuments not discoloured—the belly more tense and tender—countenance rather more anxious—pulse full—skin moist—bowels not opened—dyspnœa relieved. The operation was instantly proposed to the patient, acceded to, and performed by Mr. Babington in the unavoidable absence of Mr. Keate.

We need not describe minutely the steps of the operation; suffice it to say that a crucial incision of the integuments covering the tumour was made, and the sac cautiously opened on the director. Omentum only was exposed, and that so generally and in parts so closely adherent to the sac, that great caution was required in clearing a way for the bistoury to the seat of the stricture, that is to the neck of the sac. This was divided directly upwards by the hernia knife. The omentum not being in the least degree discoloured or injured, a strict search was made for any portion of intestine which could possibly be included within it. Such a portion of ileum doubled sharply on itself, about six inches in length, and of dark port wine colour, but not deprived of vitality, was discovered in the centre of the mass of omentum. It was drawn out, and no circular mark of a stricture seen upon it, but yet it could not be reduced without making a second incision upwards with the hernia knife, and apparently dividing some omentum. The gut was then reduced, but what to do with the unwieldy lump of omentum? To attempt to return it, adherent as it was, was out of the question; to leave it in situ was as bad. The greater portion was accordingly removed, each bleeding vessel being tied as divided. The portion lying at and adhering to the neck of the sack was left, partly to serve as a plug, partly to prevent any retraction of the remaining omentum into the belly and bleeding into that cavity. The flaps of integument were brought together by suture; lint, compress, and roller applied; and the patient, who bore the whole very well, removed to bed. The operation was necessarily tedious, but it is barely justice to Mr. Babington to mention the great caution and skill with which it was conducted. After its completion serum in some small quantity oozed from the abdomen through the wound.

Soon after the patient's removal to bed he slept quietly for three hours, and expressed himself greatly relieved throughout the night. At 4, a.m. of the 5th, he took ten grains of calomel, and soon afterwards a saline injection was thrown up. At 10, a.m. when seen by the house-surgeon, there was some dyspnœa, pulse about 116, tongue moist and clean—constant oozing of water from the abdomen, but no tenderness on pressure—no motion from the bowels.

We saw him at 1, p.m. and the symptoms had evidently altered for the worse. He was flighty, anxious, restless—pulse 100 and jerky—tongue becoming dryer and red at the tip—decided abdominal tenderness—still no motion. At 2, p.m. he was ordered:—

*Rep, hyd. sub.* gr. x. *Post horas tres ol. ricini*, ℥j.

*Hirud.* xviij. *abdomini.*

The leeches bled freely but gave no relief, the medicines were retained but procured no evacuation, and at 6, p.m. he was again evidently altering, and that rapidly for the worse. The flightiness had merged into delirium, the tenderness of the abdomen into decided pain increased by the slightest cough, which he hushed and smothered. Pulse 130, small, inclined to waver—tongue dry in centre, red at tip and sides—no motion. The symptoms of sinking quickly made progress, the usual means were employed with the usual success, and the patient died at half-past ten o'clock that evening, exactly twenty-four hours after the operation.

*Sectio Cadaveris.* Body enormously loaded with fat—legs still œdematous—face stained with stercoraceous vomit.

*Abdomen:*—Patches of inflammatory injection in peritoneum lining abdominal parietes and viscera—very little serum—a little lymph, and that only in pelvis. No intestine included in the sac—amongst the convolutions of the ileum, a dark portion about six inches long, evidently that which had been liberated from the hernia. It was bent sharply and abruptly on itself, and the contiguous sides were firmly united by recent lymph. In consequence of the abrupt angle thus made by these two portions of the adherent gut, the channel for the passage of its contents was much narrowed by the kind of *épuron* or buttress, formed by the united parietes alluded to. The peritoneal and muscular coats of the intestine were much inflamed and very dark, the mucous was very little altered. The inflammatory appearance of the outer coats was most intense at the point where the gut made the turn upon itself, and from that it gradually passed away.

Nothing particularly deserving of notice about the omentum or the sac; the stricture had been freely divided. Inguinal hernia of the common kind; sac at present empty. Liver pale, soapy, and that of a dram-drinker—kidneys, &c. pretty healthy.

*Thorax.* No fluid in pleuræ—universal, but old adhesions of left. Inferior part of either lung, especially left, crepitating very imperfectly, more solid and heavy than natural, but not hepatized. Greater part of the lungs excessively gorged with blood and serum.

Pericardium natural. Heart of enormous size, but, consideratis considerandis, very little loaded with fat. Increase of bulk mainly dependent on hypertrophy of the left ventricle unattended by any corresponding dilatation of cavity. The parietes of the ventricle in some places from an inch and a quarter to an inch and a half in thickness; the septum ventriculorum full an inch and a quarter. No valvular disease. No dilatation of the arch of the aorta. Descending, thoracic, and abdominal aorta presenting a morbid condition of the coats to a considerable extent. Very little bony but principally atheromatous deposites, with fissures and ulcerations of the inner coat. These appearances, as commonly happens, most distinct near the division into the common iliacs.

*Cranium.* Not examined.

The case has run to some length, and yet we think it is not a line longer than it should be, if any service is to be derived from it. It is interesting and important to the surgeon, as illustrating the impotence of the knife, be it ever so well handled or ever so judiciously employed, in cases where the circulating or respiratory organs are unsound. We are firmly convinced that many a bloody deed is performed especially upon the arteries, when, if the surgeon were better acquainted with the *general* facts of morbid anatomy, and especially if he were versed in the current medical literature and medical knowledge of the day, he would wash his hands, not *after* the operation but *of* it. This is a consummation which must come at last, but cannot come yet; it can only be achieved by the gradual improvement of professional education; it will probably hardly be the boast of the next generation, nor the next generation's sons. The case is likewise interesting to the physician as respects the pathology of that scapegoat for all kinds of organic diseases, asthma. Here we see a patient labouring under asthma, treated for asthma, and found after death to be affected, with what? Enormous hypertrophy of the heart. Could such have been cured, nay, would it not have been aggravated by the ordinary farrago of anti-asthmatics—emetics, æthers, and so forth? Asthma seems to be the favourite Hesperia, the unknown pathologic region of the old school; and like that sweet land of the poetic Greek, it still disappears with the march of knowledge, far, far beyond "the deep ocean" of inquiry. Several other points connected with this case are worthy of attention, amongst which we may particularize the removal of the mass of the protruded omentum. These we leave to the consideration of our readers.

II. INTERESTING CASE OF PNEUMO-THORAX, FOLLOWED BY EMPYEMA AND PERICARDITIS.

The following case is one of much interest, not only in a pathological point of view, but from the aeriform effusion into the chest having been recognized on the first inspection of the patient during life. The valuable chapter on pneumo-thorax in Laennec's work, contains a considerable number of cases of this kind, and the observations of that great man have proved in a satisfactory manner, that the disease is far more common than others which are better understood. The knowledge, however, of this, and indeed of many affections of the chest, is yet so much in its infancy in this country, that any contribution to the store of *authentic* facts must be attended with essential benefit to the profession.

On the 11th of November last, the admission-day at St. George's Hospital, Dr. Chambers, who had been examining a patient in the ward, informed several gentlemen in the waiting room, that he had just discovered a case of pneumo-thorax by percussion, and without the aid of the stethoscope. We immediately proceeded to the patient's bed, and the following are the particulars we there ascertained.

George Canning, about 24 or 25 years of age, a gardener from Richmond, admitted Nov. 11. He complains of pain in the inferior part of the *right* side of the chest, increased but not to a violent degree upon inspiration—cough without much expectoration—imperfect expansibility of right side of chest—decubitus on *left* side, and rather an indisposition to the head being low. Pulse rather quick—skin disposed to moisture, and cheeks suffused with a hectic flush—slight tendency to perspire at nights—tongue white—some thirst—bowels open from medicine.

The lower part of the right side of the chest appears to be bulged out, and nearly the whole of this side sounds remarkably hollow on percussion. On applying the ear no respiratory murmur heard below, and only a feeble one beneath the clavicle—no râle of any kind. The *left* side of the chest offers nothing particular, save that the respiration is so loud in its superior part as to be almost puerile—no unusual sound in this side on percussion.

Patient says that eleven days ago he was first attacked by the pain in the *right* side, for which he did nothing until five days after its commencement. He then applied to a medical man by whom he has been bled three times, blistered once or twice, and leeched and purged with some relief. Has been subject to cough without pain in the side throughout the Summer. The nocturnal perspirations have only been present since the 8th or 9th; he knows no cause for his illness.

From the preternatural sonorousness of the right side of the chest and absence at the same time of the respiratory murmur, we had no hesitation in believing, with Dr. Chambers, that the case was one of pneumo-thorax. At the time of our examination there certainly was no *tintement metallique*, but the absence of this did not necessarily imply the absence of the disease of which it is commonly the sign, as all, who are acquainted with the works of Laennec, are aware. In the afternoon of the 11th or morning of the 12th, the patient was again examined by Dr. Hewett with much care, and in addition to the auscultic indications which we have noticed, Dr. H. distinctly heard both the metallic tinkling, and the bourdonnement amphorique or "utricular resonance." Thus, if any faith was to be placed in auscultation, the existence of pneumo-thorax with fluid in the pleura and a communication of that cavity with the bronchia were established. The patient was ordered by Dr. Chambers,

℞. *Hyd. sub. Pulv. ant.* āā. gr. iv. *omni nocte. Haust. sal. c. Tr. digital.* ♏ viij. 6*tis hor. diæta lactea.*

On the 13th a blister was applied to the right side. We learnt on that day from the patient's friends that he had long been subject to cough, and had suffered several times from severe hæmoptysis, circumstances which, conjoined with the symptoms we have enumerated, seemed to indicate the existence of tubercles and phthisis pulmonalis. The suspicion thus excited was extremely unfortunate in its effects, for by leading all parties to view the case as one of consumption, the pneumo-thorax ceased to attract much attention. For our own parts, to our shame be it spoken, although we saw the patient afterwards on many occasions, we never re-applied the instrument. Dr. Hewett too who examined the patient, as we have mentioned, in the first instance, had no opportunity of repeating his exploration, being informed that the case had been dismissed. The subsequent notes of the case are chiefly extracted from the clinical book of Dr. Chambers.

On the 16th the digitalis was omitted, and on the 18th, compound squill pill at night, with infusion of roses and sulphate of magnesia thrice daily, were substituted for the former medicines. On the 25th, half a drachm of the spir. æth. sulph. comp. was added to each draught, and on the 4th Dec. a change in the symptoms was observed. The patient "expanded both sides of the chest nearly equally," but *could not* lie on the *left* side, which was now become the seat of pain. The pulse was very small and frequent, skin cool and not heated at night, tongue whitish, bowels open, urine free, appetite indifferent.

*Emp. Canth. lat. sinist.—Pergat.*

5th. Acute pain in left side increased on coughing or inspiration—pulse quick and sharp—skin hot—face flushed—fever — *orthopnœa* — distress. These symptoms were considered to indicate one of those attacks of pleurisy so common during the progress of phthisis. We shall see by and bye that they probably denoted something more.

*V. S. ad* ℥xij. *H. Sal. c. Vin. Ant. Tart.* ʒss. *Tr. Digital.* ♏x. *4tis horis. Omr. alia.*

The blood abstracted was buffed and cupped, the pain in the side relieved but not removed, and on the 7th Dr. C. found the upper part of the left thorax resonant, the lower not so. Six minims of laudanum were added to each saline draught, and the *pleurisy* appearing still to hang about him, he was ordered on the 10th to take two grains of calomel every night. The breathing now grew very hurried, the patient was generally observed to be sitting up, and emaciation was rapidly increasing. On the 14th the digitalis was omitted, and on the 25th, the patient complained of pain along the margin of the right false ribs, stretching thence to the hypochondrium; he continued to lie on the *right* side—the pulse was 96—the skin cool. On the 23d a blister was applied to the right side, and on the 30th another was placed upon the sternum. On the 4th of January there was a revival of the pain in the left side, and this was once more attacked by a blister. On the 8th, the bowels being much purged, the calomel pill was omitted, and next day the cough being very troublesome, he was ordered to take only spermaceti draught with squills and laudanum. The diarrhœa grew more distressing and was met with chalk mixture, &c. emaciation and debility made rapid progress, and on the 19th of the present month, January, the poor fellow died. We may observe that the most prominent symptoms latterly were the attacks of pain in the chest, the cough, and the emaciation. The expectoration was never very abundant, and at the worst only *suspicious* in its character. For the fortnight or three weeks preceding his death, the patient was generally observed sitting up in bed, and in that position he actually died These facts are deserving of attention because they do not square with the symptoms of phthisis in its latter stages.

*Sectio Cadaveris*, 20th. 2 p. m.—Body much emaciated — on percussing right side of chest, a hollow, and, as some thought, metallic sound superiorly.

THORAX.—Very considerable quantity of turbid puriform fluid in right side of chest—pleuræ themselves thickened and lined by a dense exudation, half lymph half pus—pleura pulmonalis remarkably corrugated and puckered.

*Some air in the cavity of the pleura which escaped on opening it.*

Lung remarkably compressed and condensed, *fleshy* when felt, and not crepitating at all. Its length about 6 inches, its breadth about 3, its depth from before backwards not above 2½ at the broadest part. The lobes all matted together, and the lung itself thrust to the back and central part of the pleural cavity by the pressure of the air and fluid.

At the inferior and posterior part of the upper lobe of the lung an opening through the pleura pulmonalis, about the size of a pea, with rounded and fistulous edges. Air blown into the trachea with bellows issued through it.

On laying open the bronchus, the great branch passing to the upper lobe was clearly traced by Dr. Wilson to communicate with the fistulous opening into the pleura. The channel of communication was sufficiently wide to admit the feathered end of a common writing pen, which is left in situ in the preparation.

*Pericardium* and its contents pushed preternaturally to the left by the effusion into the right side of the chest. A considerable quantity of somewhat bloody effusion into the cavity of the pericardium, and organized lymph deposited on both its surfaces. Heart little, if at all, enlarged in any sense—valves and great vessels healthy.

*Pleuræ* on left side generally adherent by rather recent lymph—no fluid.

Throughout the left lung, which was large, several groups of transparent miliary tubercles, unaccompanied by consolidation of the contiguous parenchyma. At the apex of the lung these tubercles were more numerous; several of them larger than pins' heads, yellow, and opaque, in short beginning to maturate; none of them softened; not a vomica to be found. Many of the tubercles situated immediately beneath the pleura pulmonalis.

On the whole the tubercles might be said to be few in number, in their earliest stage, and not materially interfering with the function of respiration.

ABDOMEN:—Liver lower and more to the left than natural, from the effusion in the right side of the chest; nothing further of any consequence observed.

CRANIUM:—Not examined.

The present case, when taken in connexion with that of Mr. Cornish, published by the Editor of this Journal, is worthy of consideration, and attentive consideration too, on the part of the profession. Happening in this town within a few months of each other, they shew the not unfrequent occurrence of a disease, the very name of which is a puzzle, its existence a problem, to many of the practitioners in London. The reason is clear;—that without auscultation or percussion, it CANNOT be recognized during life, it probably will not be discovered after death. In Dr. Johnson's case, as our readers well know, the operation of paracentesis thoracis was performed. What would have been its effect in the present instance? This can only be conjectured from taking a review of the progress of the case. A patient of scrofulous aspect labours long under cough and occasionally has hæmoptysis; all at once symptoms of acute pleurisy in the right side of the chest supervene; and in eleven days from the commencement of this attack, being examined by the stethoscope, he is found to labour under the symptoms of pneumo-thorax, liquid in the pleural cavity, and a communication with the bronchi. Is it not rational to conclude that this patient had tubercles in the right lung; that one of these tubercles, probably situated immediately beneath the pleura, gave way; that the atmospheric air escaped from the bronchi into the pleural cavity through this opening, and that, being there, it gave rise to pleurisy and its consequence, effusion. It is not only rational to conclude that such was the case, but it seems to us impossible to do otherwise.

Now, having brought the patient to this point, let us carry him to the close of his career. After awhile he is attacked by pain and the symptoms of inflammation, first in the left side, then again in the right, then beneath the sternum, and, finally, in the left side once more; he emaciates rapidly; spits comparatively little, and dies. On dissection, in addition to the empyema and pneumo-thorax on the right side, there are found the evidences of chronic pericarditis, pleural adhesions on the left side, and a few crude tubercles in the left lung. Is it not evident that the inflammation of the pericardium and left side was merely the propagation and extension of the matrix, as it were, that existed on the right, the nidus and focus of mischief? Is it not evident that the patient did not die of phthisis, strictly speaking, at all; but of the empyema and pneumo-thorax in the right side, and their consequences elsewhere? The proposition would seem to be incontrovertible. The question then comes to be, would the operation of paracentesis thoracis have relieved him? As far as the actual causes of his death were concerned, it undoubtedly would have done so. There are two things, however, to be taken into the account in the investigation of this interesting and important point. In the first place, the patient might die of the immediate effects of the operation, a circumstance which, in logic as well as in common sense, would interfere with the success of the measure. This objection, however, is equally applicable to the operation for empyema simply, and yet it is allowed to be, cæteris paribus, proper in that disease. In the next place, the pneumo-thorax, which gave rise to all the rest, was itself, if our reasoning be correct, the consequence of tubercles more or less advanced. Now tubercles in the lungs, or phthisis pulmonalis, we know to be a very fatal, if not an incurable disease; ergo, it follows as a fair deduction, that even if our operation be successful and cure the pneumo-thorax, it still leaves behind it an incurable or nearly incurable malady, viz. tubercular phthisis.*

The operation, then, not holding out a very cheering prospect in this kind of pneumo-thorax, we are led to inquire whether it bid more fairly in any other form of this affection. We believe that it does so in that variety not complicated with an aperture into the lung. Into this field we may not enter, but refer our readers, as we have done before, to the admirable work of Laennec.

---

XIII.

* If, however, the pneumo-thorax in itself be the cause of extreme distress, and probably of rapid death, as in Dr. Johnson's case, we conceive that all such arguments as these must merge in the indication of giving present relief and disregarding possible consequences. No man of science and courage would stand tamely by, and see his patient die like a dog before his face without an effort to save him. Logic here must yield to something higher than logic—humanity and moral daring.

### CLINICAL REVIEW.

### LIII.

### ST. GEORGE'S HOSPITAL.

### On the Connexion between Pulmonary Apoplexy and Contraction of the Left Auriculo-Ventricular Opening, with Hypertrophy of the Right Side of the Heart.

It is a curious, and in a pathological point of view, a very interesting fact, that when the left auriculo-ventricular opening of the heart becomes straitened and contracted, in consequence of disease of the mitral valve, pulmonary apoplexy is a frequent result. When a constriction of the channel between the left auricle and ventricle may be considered as commencing or established, a series of actions and changes are induced, which illustrate in a remarkable manner the effects of the vis medicatrix naturæ of Cullen, and the mischief to which that reparative process will give rise if it fail in its sanative object. This is perfectly consistent with what commonly happens in the animal economy, for it is undeniable that the efforts of the physician or surgeon are much less frequently directed to the counteraction of disease per se, than to meeting and combating the injurious excesses and results of that boasted vis medicatrix. Without it, indeed, we could do nothing, for it gives us tangible phenomena to struggle with, and is to us what a fulcrum would be to Archimedes *could* he move the world. Thus,

> Dear Nature is the kindest mother still.

But to revert to the contraction of the left auriculo-ventricular opening, or ostium arteriosum as it is sometimes denominated. As soon as this is established to any extent, an additional onus is thrown upon the right ventricle, which it meets, as all muscles do, by an increase of power and bulk. It would be difficult to determine whether the first stage of this enlargement be dilatation of cavity or hypertrophy of parietes, but certainly after a time we meet with both, and the right side of the heart affords an example of what English practitioners vaguely term active enlargement, and is more precisely designated by the French, hypertrophy and dilatation. As long as this augmentation of volume is kept within moderate bounds it is to be looked on as an effort of the vis medicatrix, and contributes to the equalization of the circulation and the safety of the individual. But the constriction and impediment to the passage of the blood through the left side of the heart being constantly on the increase, an incessant call is made upon the muscular power of the right ventricle and its enlargement proceeds pari passu, till at length the time arrives when this of itself becomes a morbid condition, and what was at first a reparative process is doomed at last to be the cause of the destruction of the patient.

It must be remembered, that the capillary system intervening between the circulation of the right and the left side of the heart, is peculiarly delicate in every respect. It consists of the fine and extremely minute ramifications of the pulmonary artery and veins, and is situated in an organ of remarkably porous and very fragile texture, as compared with the grosser and tougher tissues of the trunk and the extremities. And yet this system of vessels and this frail texture of the lungs are to bear the brunt of the morbid increase of power in the right side of the heart, the progress of which we have attempted to describe. Nay, not only is there a hypertrophied right ventricle pumping the blood with unnatural violence into the pulmonary organ, but this is prevented from unloading itself or being unloaded, by the difficulty with which the fluid is propelled through the left auriculo-ventricular channel. The inevitable consequence is, that a perpetual congestion must exist in the lung, its vessels must be always gorged, an unceasing strain must be maintained on the small branches of the pulmonary artery, at length these vessels give way, the blood is extravasated in the texture of the lung, and pulmonary apoplexy is produced.

We know that some have ridiculed the term "apoplexy of the lungs," but provided that the nature of the affection be definitively understood, we need care little for verbal disputes. Words are only of value as expressing things, and if the established mode of nomenclature were reversed, and what is now called a hat were named a brick, and a brick called a hat, we really conceive that it would signify little, provided every one knew what was meant by the expressions. Apoplexy of the lungs, then, implies an extravasation of blood into their texture, which may or may not be attended with hæmoptysis, but is not essentially connected with it. It has been a question, whether the blood be extravasated into the proper cellular tissue of the lung, or into the ultimate terminations of the bronchi, constituting the air-cells. This is a point which we really cannot pretend to determine, our eyes not being microscopes, and even microscopes not furnishing conclusive evidence. We believe, however, that in this, as in the effusion of lymph in peripneumony, constituting hepatization, the deposite is both in the cellular texture and the air-cells; but this is a pure speculation, and may be taken for as much as it is worth.

When we pass to more obvious anatomical facts, we remark that when the extravasation has been present for a certain length of time, it presents a very curious appearance. The lung is more or less studded with blocks, nearly square, of very solid and hard material, of the colour of a clot of blood. Sometimes these are immediately beneath the pleura pulmonalis, sometimes deeper in the pulmonary texture; the boundaries of every block are remarkably defined, and evidently formed by the interlobular partitions of cellular membrane, which divide each lobe into many compartments; the lung in the neighbourhood is commonly quite healthy, but occasionally there is a kind of gradation observed between the perfect apoplectic mass and the natural parenchyma; and, finally, no blood whatever is in general discovered in the bronchi. On making a section of a block, it presents the deep reddish-black colour of old coagulum of blood, shews a very firm and clean-cut surface, and has the granular texture observed in a hepatized lung. This is the condition of the apoplectic extravasations some time after their first formation. Of course they would present a very different appearance if seen at their commencement, but as such an opportunity has never occurred to us, we cannot speak from our own observation on the subject. M. Laennec, who gives an admirable description of the disease, takes no notice of its earlier stages, save in one short paragraph, where he mentions that abundant "exhalations of blood" into the lungs will sometimes produce sudden death; and on examining the bodies of such patients, more or less extensive clots of blood are perceived in the middle of the lacerated lung, precisely resembling apoplectic extravasations into the cerebral tissue. In a case of this kind, related by M. Corvisart, the effusion was so great, that the lung and the pulmonary pleura had been ruptured, and the blood had escaped in quantity into the pleural cavity. Before we quit this subject, we would caution our readers not to mistake the effusions of blood which take place in small vomicæ in diseased and tuberculated lungs for instances of pulmonary apoplexy; a mistake which we have seen occur more than once, and which an ill-informed or superficial observer might readily commit.

There is another point, in the pathology of cases of contraction of the left auriculo-ventricular opening, to which we would allude, before we proceed to the consideration of the cases themselves. It is this, that, nearly in proportion to the growth and enlargement of the right ventricle, is the flaccidity and almost atrophy of the left. The reason is obvious, for the very obstruction that created a demand on the muscular power of the one, prevented the exercise of that of the other, and that which produced the augmentation of the former, by the same law of nature tended to the wasting of the latter. As the left ventricle grows small and feeble, the aorta and the whole of the aortic system also degenerate, and that, in some instances, to a very remarkable degree.

Having made these preliminary observations, which we think were required for the right understanding of the disease, we shall now relate the particular facts on which they have been founded.

*Case* 1. Mary Hawkins, ætat. 41, admitted Sept. 2d, 1829, under Dr. Seymour.

Face puffy, and leuco-phlegmatic, with a bilious tint—lower extremities œdematous—apparently some ascites—extreme orthopnœa—lies best on right side in consequence of "something falling over in her inside" when she turns to the left—liver felt enlarged, and the margin of its right lobe very sharp and distinct—some cough, with little expectoration and *no hæmoptysis*—palpitations—no pain in chest—dyspnœa on exertion, which indeed is impossible—distressing flatulence.

Pulse *small* and frequent—tongue moist—bowels open—urine scanty and high-coloured.

Has laboured under more or less dyspnœa for the last twelvemonths, increased by exertion and especially by ascent; palpitations for about the same length of time. Two months ago was confined, after going her full time, and the child was born alive but is since dead. Much flooding followed delivery, and she dates her orthopnœa and more urgent symptoms from that period. Has suffered for some time from pain in the epigastrium and region of the liver.

On placing the hand over the præcordia, a sensation of extensive though not powerful action is communicated to the fingers, and on making percussion the dull sound of the heart is more extensive than natural. On applying the stethoscope to the præcordia, the heart's action is heard over all the left, most of the right thorax, and even above the clavicles—both auricular and ventricular sounds loud and clear—ventricular accompanied with more than usual impulsion. The ventricular systole and pulse at the wrist *do* correspond, *but the latter is remarkably weak in comparison.* No bruit in the aorta.

State of lungs not examined in consequence of the great dyspnœa.

Diagnosis. *General dilatation of the heart—dilatation, with some hypertrophy, at least no diminution of the parietes of the ventricles.*

The patient was ordered a quinine draught twice daily, with aloes and squills at night, and cream-of-tartar drink. She sank quickly and died on the 5th, three days after her admission.

*Sectio Cadaveris* :—Legs œdematous —face puffy and of bilious hue.

*Thorax.*—Two or three pints of clear serum in right pleural cavity, compressing the lung into the top of the chest, and thrusting the diaphragm and liver downwards. Little fluid in left pleural cavity, and no adhesions.

Right lung greatly compressed by the fluid, carnified in some parts, gorged with serum in all. It presented two or three specimens of pulmonary apoplexy, one as large as the square pieces of Castile soap which are commonly sold in the shops. Left lung more respirable than the right, but œdematous and gorged with blood; it also presented some examples of pulmonary apoplexy.

Heart externally very large. Cavities of right auricle and ventricle greatly dilated—cavity of left auricle also dilated. Left auriculo venticular opening much contracted by approximation of the two alæ of the mitral valve, which were not ossified, but extremely thickened and indurated, with some brittle *végétations* attached to them. Cavity of left ventricle and parietes nearly natural, but if any thing, rather diminished in volume.

No induration of semi-lunar valves, but slight and easily separable *végétations* annexed to them—aorta not dilated, but presenting incipient steatomatous depositions.

In the appendix of the right auricle a laminated coagulum like that of aneurism. In the left auricle, coagula so large and dense as completely, or nearly, to fill the chamber. This polypus was clearly of some standing, being brown, not black, in colour, more or less laminated in structure, and containing in its centre a cavity filled with purulent-looking matter. Many smaller coagula in the appendix had cavities in their centre, containing matter like putrid pus. No injection of inflammatory appearance of the lining membrane of the heart.

*Abdomen ;*—A small quantity of clear serum in its cavity. Liver descending low, and its right lobe presenting a remarkably sharp crescentic edge; its structure indurated, and resembling a nutmeg in appearance. Kidneys pretty healthy.

Case 2. Elizabeth Martin, a married woman, from Surrey, æt. 35, admitted Sept. 16, 1829, under Dr. Chambers.

Anasarca of lower extremities—ascites—enlarged and indurated liver—pain in the right hypochondrium—palpitations of heart with venous undulation in the neck. Pulse 120, small, regular—skin cool—tongue white—bowels open—urine scanty and high-coloured.

Ill two years—much worse within the last two months—principal distress has been from dyspnœa and palpitations.

She was ordered mercurial inunction with nitre and diuretic medicines, but under these means she became much lower, and effervescing draughts with sp. æth. nit. were substituted. Aphthæ appeared upon the mouth and cheeks, she was troubled with vomiting, and on the 29th we find the following report :—

Says that yesterday afternoon she was attacked with pain in the right side of the chest and aggravation of dyspnœa. The pain has nearly subsided, but the difficulty of breathing remains with a frequent cough, and expectoration of froth and *blood.* Pulse not so feeble as it has been—skin moderately warm. *Hirud.* xx. *pect.* *Resumatur Haust. Nitri* c. *Vin. Ipec.* ℈ss. *4tis. horis.*

30th. Breathing relieved — orthopnœa—sputa less blood stained. Sensation of burning all over her abdomen and up her throat, but pressure there does not cause pain—pulse very soft and small—tongue still aphthous.

A demulcent draught was prescribed, but the prostration increased, the burning sensation alluded to grew more severe, diarrhœa set in, and on the 1st of October the patient died.

*Sectio Cadaveris.* Lower extremities anasarcous—body not deficient in fat.

*Thorax.* Very great quantity of yellow serum in right side of chest, with lymph, partly recent, partly old, on the pleuræ, and irregular intervening bands. Same appearances but not to so great a degree on the left side.

Right lung compressed, as it were into a corner by the fluid; crepitating, and that imperfectly at the apex only; and studded with apoplectic extravasations of blood, intermixed with portions of parenchyma nearly colourless but remarkably solid and hard. Left lung in a similar condition, but rather more respirable than the right.

Some clear fluid in the pericardium. Heart extremely large. Cavities of right auricle and ventricle and of left auricle much dilated, and their parietes hypertrophied; hypertrophy of right ventricle particularly remarked. Cavity and parietes of left ventricle on the whole little altered.

Valves on right side healthy—*pulmonary artery dilated.* Mitral valve thickened, and its aperture considerably straitened—its chordæ tendineæ very thick. Semilunar valves of aorta thick and indurated. Aorta itself *very small,* with slight and incipient steatomatous deposites.

*Abdomen.* Little fluid in this cavity. Liver enlarged, hard, of a nutmeg appearance, and gorged with black blood. Spleen small, but hard. Kidneys hard but otherwise healthy. Small serous cysts connected with the ovaria.

*Cranium.* Much clear serum between arachnoid and pia mater—veins rather turgid—more serum than usual in ventricles.

In the foregoing case, the stethoscope, as far as we know, was not applied; it was supposed to be one of enlargement of the heart, but the precise nature of the disease was not suspected. In the following instance, the diagnosis drawn from the stethoscopic signs will be found to be tolerably exact.

Case 3. William Lambert, æt. 52, a green-grocer, admitted Sept. 13, 1829, under Dr. Chambers.

Cough—expectoration of viscid mucus, much stained with arterial blood—pain under sternum—dyspnœa—indisposition to lie on right side, which he is said, in Dr. Chambers' book, to expand most in inspiration—belly swelled and ascitic—liver enlarged and indurated — legs slightly œdematous. Pulse 70, small, weak, regular—skin cool and dry—tongue red, clean, moist—bowels open from medicine—urine scanty and high-coloured.

Attacked with cough and dyspnœa about Christmas last — abdomen has been swelled on or off for two or three months; the legs for about a week.

Cupping, diuretic medicines with purgatives, and mercurial inunctions, were the means employed, but being purged, and becoming very low, he was allowed a gill of gin in water daily. On the 22d, when we first examined him, we made the following report.

Cough very troublesome, especially at night, when he spits much frothy mucus, stained with blood—distressing pain across lower end of sternum increased by a full inspiration, which excites cough—breathing extremely short—orthopnœa—decubitus difficult on either side, but he prefers lying on the left—night delirium—distressing flatulence. Pulse very small, but regular and not rapid — skin cool — face not swelled — tongue yellowish, loaded — bowels open—urine scanty and high-coloured.

Pulsation in epigastrio and under left nipple, but not violent. On using percussion, an extensive dull sound in the cardiac region—sound pretty good on upper and anterior part of left thorax, not so good behind and below. Sound indifferent in right thorax. On applying the stethoscope, respiration loud on either side of chest below clavicles, more obscure and bronchial below, especially on the right side.

Heart's action heard extensively, especially in a transverse direction, and very audible below *right* clavicle—sound of ventricle dull—little impulsion, but most in the situation of the right ventricle—no bruit—no irregularity.

DIAGNOSIS. *Effusion into either side of thorax, particularly right—pulmonary apoplexy?*

*No hypertrophy of heart, unless the right ventricle be somewhat increased in the power of its parietes—dilatation of cavities.*

*Certainly obscurity about the case.*

He made no improvement and a large blister was applied, and the ulcerated surface dressed with mercurial ointment. On the 3d of October the blister was repeated, but he gradually sank, and finally died on the 13th. The medicinal treatment chiefly consisted of anodynes.

SECTIO CADAVERIS:—Body much emaciated—skin sallow.

*Thorax.* Nearly a gallon of clear yellow serum in right pleural cavity—pleuræ themselves injected, and coated with false membranes. A little fluid in left side, without any lymph.

Right lung compressed to one-third its natural size, and tucked up into the top of the chest—inferior lobes *carnified*,* and quite impervious to air. Upper lobe not so destitute of crepitation. At posterior and inferior part of lung a block of rather ancient apoplexy, with a kind of circumvallation of hepatized lung around it.

Left lung gorged with serum in its lower lobe—at inferior and anterior part a square block of apoplexy.

No fluid in, nor adhesions of, the pericardium. Heart of enormous size—auricles and right ventricle much dilated with little alteration, but no diminution, in the parietes of the latter. Cavity of left ventricle quadrupled at the least, and its parietes so thin, that at one part only the lining membrane and pericardium remained. The internal membrane presented steatomatous deposites, similar to those of the arterial tunics, and in many places it was abraded like them by ulceration; to these ulcerated parts old laminated coagulum adhered. Opposite the thinnest part of the parietes of the ventricle, a mound of organized lymph had been laid on the pericardium investing the heart, evidently for the purpose of protection.

Valves quite healthy—arch of aorta rather dilated.

*Abdomen:*—Some fluid—liver enlarged, hardened, and of nutmeg appearance. Other viscera healthy.

*Cranium:*—Not examined.

In this case there was no disease of the mitral valve, but the extreme dilatation and tenuity of the left ventricle as compared with the right, the parietes of which maintained their natural force, destroyed the balance of power, and threw the weight of it into the scale of the right side of the heart. The consequences in a physiological point of view were nearly the same as if the left auriculo-ventricular passage had been obstructed; the lungs were exposed to an undue degree of violence, and pulmonary apoplexy ensued.

CASE 4. Examined Dec. 2d, 1829.

A stout, athletic, and apparently middle-aged man, received a fall or a violent injury, was brought into the hospital, and died in some six and thirty hours, apparently from internal hæmorrhage.

On opening the body the cause of death was found to be an extensive rupture of the spleen and effusion of blood into the cavity of the peritoneum. We were occupied at the time in opening another body, but after the examination of this patient was completed, and the gentlemen operating had disappeared, we were struck by the size of the heart which had been exposed but left untouched.

It was at least twice as large as na-

---

* Carnification differs, toto cælo, from hepatization in this, that it is not the result of inflammation, but of compression from fluid, &c.

tural. The right auricle greatly dilated and its musculi pectinati preternaturally strong; the right ventricle not much dilated, but decidedly hypertrophied, the increase of power being chiefly, as usual, in the columnæ carneæ. *The pulmonary artery was large.* The left auricle dilated and somewhat hypertrophied. The left ventricle was neither dilated nor hypertrophied, but flaccid in its parietes, and its columnæ were positively smaller than those of the right.

The left auriculo-ventricular opening was so narrowed as to form a kind of rima or chink an inch in length, and scarcely more than two lines in breadth at its widest part. The limbs of the mitral valve and contiguous portion of the internal tunic of the auricle were corrugated, thickened, and roughened by sharp, firm, bony scales.

The lungs were so destroyed that we could only collect portions of them. There were old adhesions of the pleuræ in many places—a disposition to carnification—and in one place a small but genuine specimen of pulmonary apoplexy.

This patient had no dropsy, and of his history we know nothing, but it is not a little singular that with disease in so advanced a stage and of so serious a nature, he should still have been capable, as to all appearance he was, of following a laborious occupation. The only other example of this disease which we have hitherto had an opportunity of witnessing was in the case of a female patient of Dr. Wilson's at the hospital. We did not see her during life, but she was considered by Dr. W. to labour under organic disease of the heart. On dissection there were found:—apoplexy of the lungs to a considerable extent—general enlargement of the cavities of the heart, excepting that of the left ventricle—hypertrophy of the right ventricle—much narrowing of the left auriculo-ventricular opening and puckered bony condition of the mitral valve—flaccidity and atrophy of the left ventricle.

In a small but beautiful collection of preparations belonging to Dr. Hewett, is a specimen of this contraction of the left auriculo-ventricular opening. Appended to the preparation is a note of the case and the dissection, in which it is stated that several solid square blocks of "hepatization" were discovered in the lungs. Upon consideration, Dr. Hewett is of opinion that these were in reality instances of pulmonary apoplexy, an affection which was not at the time of the examination sufficiently understood in this country.

M. Cruveilhier, in the third livraison of his valuable series of plates on morbid anatomy, mentions the particulars of a case of extensive apoplexy of the lungs, conjoined with contraction of the left auriculo-ventricular opening.* In that instance the French pathologist states that the *left* ventricle was hypertrophied, and he refers to a case of M. Andral's in which hypertrophy and dilatation of the left ventricle existed, without any affection of the mitral valve or left auriculo-ventricular opening. In M. Andral's case, however, the aorta was remarkably small. These are all the cases of this interesting affection that we have witnessed, or to which we can refer at present, although we must confess that we have not consulted many authors on the occasion. We think that the report of the four or five cases from St. George's Hospital, occurring within so short a time of each other, is a better proof of the comparative frequency of the disease, than any laborious quotations or researches.

The symptoms must be considered as very obscure, but still we think that by combining the general phenomena of the disease with the hints supplied by the stethoscope, we may perhaps be enabled to form a pretty just suspicion.

In general there seems to be more or less dropsy, especially in the form of anasarca of the lower extremities—dyspnœa and the other usual symptoms of organic disease of the heart—cough, and in many instances some hæmoptysis. The pulse in all the cases which we have seen, or of which we have read, has been *extremely small*,

* This case will be found detailed in the 23d Number of this Journal, page 87, et seq.

and here we may observe, par parenthèse, that the common notion of a small pulse being occasioned by disease of the semilunar valves of the aorta, is in ninety-nine cases out of the hundred erroneous. At the time that the pulse is so small, the extensively dull sound on percussion of the cardiac region indicates enlargement of the heart, whilst the action of the ventricles is more tumultuous, and their systole, especially in the right ventricular region beneath the sternum, will probably be attended with more impulsion or shock than natural. There may or may not be a *bruit*, but if the hypertrophy and dilatation of the right ventricle be considerable and the pulmonary artery large, we think that it commonly exists. If present, it will then be distinct in the region of the pulmonary artery, which is close to the left side of the sternum, between the cartilages of the second and fourth ribs. At the time that the heart is acting vigorously and the *bruit*, or increased action without a bruit, are discovered in the pulmonary artery, the *arteries* in the neck will be tranquil, and the stethoscope placed above the right clavicle will detect no impetus or unusual sound in the aorta. We think that the foregoing combination of symptoms will in most instances enable a man of tolerable sagacity and tact to recognize the disease, but as yet we know too little to dogmatize upon the subject. H. J.

